# Generation of a Mouse Model of Fuchs Endothelial Corneal Dystrophy by Knock-in of CTG Trinucleotide Repeat Expansion in the *TCF4* Gene

**DOI:** 10.1167/iovs.66.6.18

**Published:** 2025-06-04

**Authors:** Yuki Oyama, Suguru Ito, Taichi Yuasa, Mizuki Ueda, Satoshi Chiba, Tatsuya Nakagawa, Ayaka Izumi, Masahito Ikawa, Noriko Koizumi, Naoki Okumura

**Affiliations:** 1 Department of Biomedical Engineering, Faculty of Life and Medical Sciences, Doshisha University, Kyotanabe, Japan; 2 Graduate School of Pharmaceutical Sciences, Osaka University, Suita, Japan; 3 Department of Experimental Genome Research, Research Institute for Microbial Diseases, Osaka University, Suita, Japan; 4 ActualEyes Inc., Kyotanabe, Japan

**Keywords:** Fuchs endothelial corneal dystrophy, TCF4, trinucleotide repeat expansion, corneal endothelium

## Abstract

**Purpose:**

Fuchs endothelial corneal dystrophy (FECD) is frequently associated with trinucleotide repeat (TNR) expansion in the *TCF4* gene intron. The aim of this study was to establish a novel FECD mouse model with TNR expansion.

**Methods:**

We used CRISPR/Cas9-mediated genome editing to generate knock-in mice carrying 100 CTG repeats in the *Tcf4* intron. Corneal endothelial phenotypes were evaluated using specular microscopy and transmission electron microscopy. Transcriptome analysis was performed using RNA sequencing of corneal endothelial tissue from *Tcf4*^(CTG)100/(CTG)100^ and wild-type mice.

**Results:**

*Tcf4* ^+/(CTG)100^ and *Tcf4*^(CTG)100/(CTG)100^ mice developed characteristic FECD features, including progressive guttae formation and decreased corneal endothelial cell density. At 60 weeks, *Tcf4*^+/(CTG)100^ mice showed increased guttae percentage (0.314% ± 0.145%) versus wild-type (0.170% ± 0.089%), although not statistically significant. *Tcf4*^(CTG)100/(CTG)100^ mice exhibited significantly higher guttae formation (0.563% ± 0.293%) compared to controls. Similarly, endothelial cell density showed non-significant reduction in *Tcf4*^+/(CTG)100^ (1629 ± 71 cells/mm^2^) versus wild-type (1704 ± 68 cells/mm^2^), whereas *Tcf4*^(CTG)100/(CTG)100^ mice demonstrated significant decrease (1600 ± 76 cells/mm^2^). RNA sequencing identified 3221 differentially expressed genes (579 upregulated, 2,642 downregulated), with enrichment in pathways related to adaptive immune response, chemokine signaling, and cytokine-cytokine receptor interaction.

**Conclusions:**

Our study demonstrates that TNR expansion in the *Tcf4* intron, on its own, is sufficient to induce FECD phenotypes in vivo. This mouse model provides a valuable tool for investigating FECD pathogenesis and developing targeted therapeutics.

Fuchs endothelial corneal dystrophy (FECD) is the most prevalent form of corneal dystrophy,^1^^–^^3^ with an estimated prevalence of 7.33% (95% CI, 4.08%–12.8%).^4^ The disease is characterized by the formation of guttae, which are extracellular matrix (ECM) excrescences that develop between the corneal endothelium and its basement membrane (the Descemet membrane) and initially appear in the central cornea. As the disease progresses, guttae demonstrate increased confluency and expand from the central to mid-peripheral regions of the cornea.^5^^–^^7^ This progression leads to light scattering throughout the cornea and results in decreased visual acuity.^8^^,^^9^ The progressive corneal endothelial cell damage also reduces endothelial cell density. When this density falls below a critical threshold (500–1000 cells/mm^2^), the remaining endothelial cells can no longer maintain proper function, and the end result is corneal edema and severe visual impairment.^10^^,^^11^

The genetic component of FECD has long been recognized through its familial occurrence patterns, although sporadic cases suggest the involvement of multiple genetic and environmental factors.^1^^–^^3^^,^^12^ A landmark discovery in 2012 by Wieben et al.^13^ revealed that approximately 80% of FECD patients carry CTG trinucleotide repeat (TNR) expansions exceeding 50 repeats in the *TCF4* gene intron. Subsequent studies have confirmed this association across various ethnic populations, albeit with differing frequencies.^14^^–^^21^ Currently, *TCF4* TNR expansion is widely accepted as the most common genomic mutation in FECD.^2^ This pivotal discovery has accelerated research into FECD pathogenesis, leading to multiple hypotheses regarding the mechanisms of TNR-induced corneal endothelial cell damage.^22^^–^^29^ However, diseases that develop from intronic TNR expansion, which does not directly affect protein-coding sequences, have as yet unknown pathogenic mechanisms, as evidenced by the numerous pathogenic pathways that have been proposed.^2^

In the present study, we addressed the fundamental question of whether *TCF4* intronic TNR expansion alone is sufficient to induce the FECD phenotype by taking the approach of generating and characterizing a knock-in mouse model that carries this expansion. Our overall aim was to establish this disease model as a valuable tool for elucidating FECD pathogenesis and developing novel therapeutic approaches.

## Material and Methods

### Animal Studies

All experimental procedures involving animals were conducted in accordance with protocols approved by the Institutional Animal Care and Use Committees of Doshisha University (Approval No. A-23011; Kyoto, Japan) and Osaka University (Approval No. Biken-AP-H30-01; Osaka, Japan). The studies adhered to the Association for Research in Vision and Ophthalmology Statement for the Use of Animals in Ophthalmic and Vision Research. Mice were obtained from CLEA Japan, Inc. (Tokyo, Japan), Japan SLC, Inc. (Shizuoka, Japan), and Shimizu Laboratory Supplies (Kyoto, Japan) and were housed under standard laboratory conditions with a 12-hour light/dark cycle. All recombinant DNA procedures were performed in compliance with institutional guidelines and approved by the Recombinant DNA Experiment Committee at Doshisha University (Approval No. D23011).

### Generation of *Tcf4*^+/(CTG)100^ and *Tcf4*^(CTG)100/(CTG)100^ knock-in Mice Using CRISPR/Cas9

**Figure 1.**
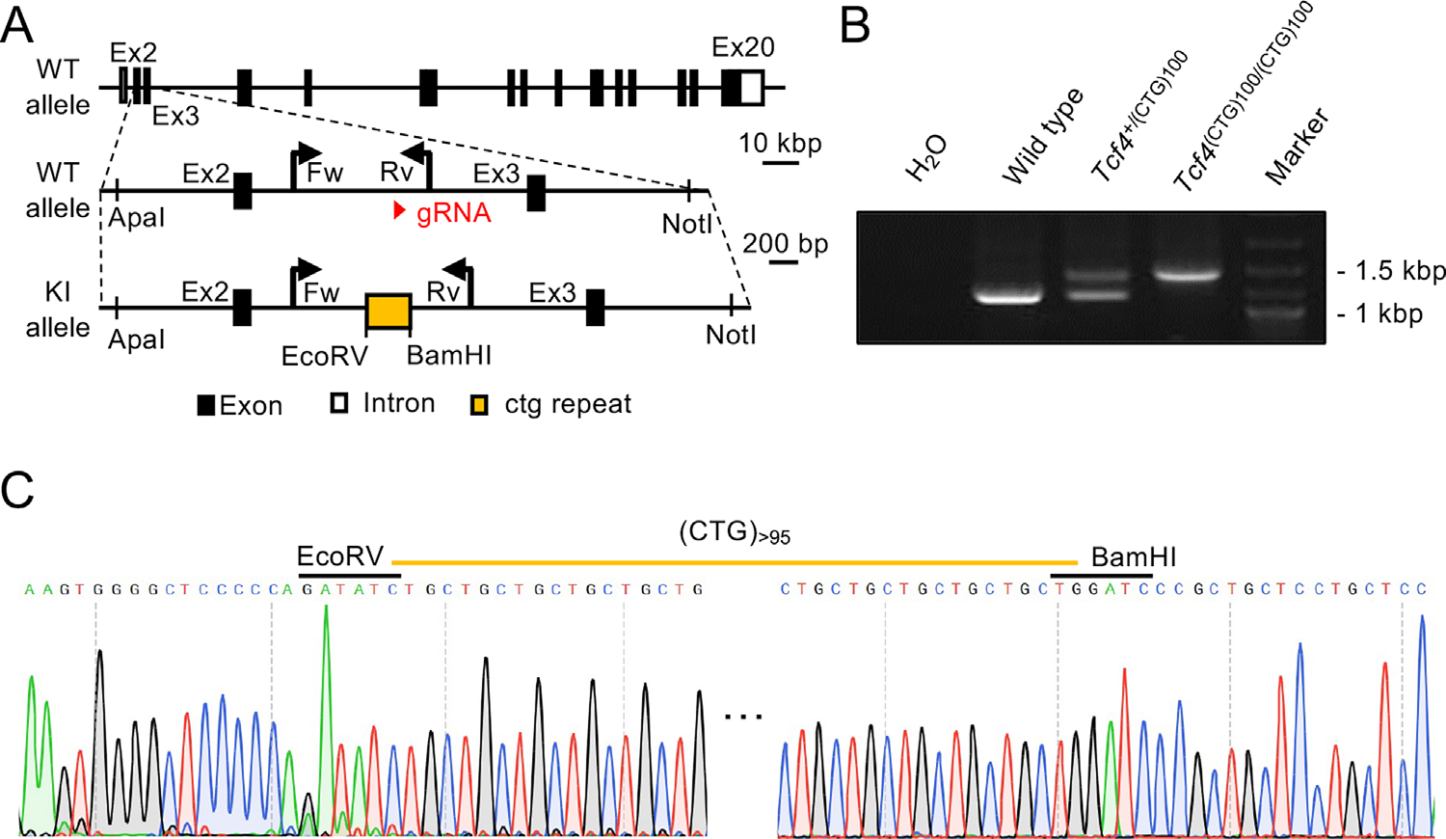
Generation of *Tcf4*^+/(CTG)100^ and *Tcf4*^(CTG)100/(CTG)100^ knock-in mice using CRISPR/Cas9-mediated genome editing. **(A)** Targeting strategy for *Tcf4* CTG repeat knock-in. The gRNA was designed to target the intron 2 region, and synthetic oligonucleotides containing 100 CTG repeats were inserted via homologous recombination. *Black boxes* indicate exons, and *arrows* show the primer positions (Fw and Rv) used for genotyping. **(B)** Genotyping of *Tcf4*^+/(CTG)100^ and *Tcf4*^(CTG)100/(CTG)100^ mice. Wild-type allele produces a 1144 bp band, whereas knock-in allele generates a 1456 bp band. Heterozygous *Tcf4*^+/(CTG)100^ mice display both bands. H₂O served as a negative control. **(C)** Sequence analysis confirming CTG repeat insertion. Direct sequencing shows successful integration of more than 95 CTG repeats at the target site, flanked by the EcoRV and BamHI restriction sites.

CRISPR/Cas9-mediated genome editing was used to generate *Tcf4* CTG repeat knock-in mice. Based on the reported position of CTG18.1 repeat expansion in FECD patients,^13^ CTG repeats were designed to be inserted in the intron 2-3 region of the mouse *Tcf4* gene. Prior to generating the knock-in mice, sequence analysis was performed, which revealed high conservation between human *TCF4* and mouse *Tcf4*, with 66% sequence identity in the intron 2-3 region, 92% identity in the open reading frame, and 98% identity at the protein level. Notably, the wild-type mouse *Tcf4* genome contains no CTG repeat sequence in this region. A plasmid expressing both Cas9 and single-guide RNA (sgRNA) targeting the *Tcf4* exon 2 was constructed using the pX459 vector (no. 48139, Addgene, Cambridge, MA, USA) (Supplementary Table S1). The homology-directed repair template was generated in pBluescript II SK (+) vector by incorporating 1.6 kb and 2.0 kb homology arms flanking the target site, along with a synthesized (CTG) 100 repeat sequence inserted using EcoRV and BamHI restriction sites. The final construct was cloned into the ApeI-NotI sites of the vector (Fig. 1A).

The gRNA design and selection were performed using CRISPRdirect to minimize potential off-target effects.^30^ The DNA cleavage efficiency of the selected gRNAs was evaluated using a reporter assay system.^31^^,^^32^ Based on these analyses, we selected gRNAs that exhibited high on-target activity with minimal predicted off-target effects.

EGR-G01 embryonic stem (ES) cells were co-transfected with the Cas9/sgRNA plasmid and the repair template following established protocols.^33^^,^^34^ Successfully edited ES cell clones were identified using PCR genotyping (Supplementary Table S1). Selected clones were injected into eight-cell stage Institute of Cancer Research (ICR) embryos, and the resulting chimeric blastocysts were transferred to pseudopregnant females, as previously described.^34^ Heterozygous *Tcf4*^+/(CTG)100^ mice were established by crossing chimeric founders with C57BL/6J mice. Homozygous *Tcf4*^(CTG)100/(CTG)100^ mice were subsequently generated by interbreeding heterozygous *Tcf4*^+/(CTG)100^ mice. Both heterozygous *Tcf4*^+/(CTG)100^ and homozygous *Tcf4*^(CTG)100/(CTG)100^ mice were used for experimental analyses. Genomic DNA was extracted from mouse tail biopsies for genotyping and sequence verification of CTG repeat insertion in the *Tcf4* gene. Genotyping was conducted using PCR with KOD FX Neo polymerase (TOYOBO, catalog number: KFX-201; Osaka, Japan). PCR amplification for sequence verification was conducted using specific primers (Supplementary Table S1). The PCR products were purified using the Wizard SV Gel and PCR Clean-Up System (Promega Corporation, Madison, WI, USA) before Sanger sequencing analysis.

### Preparation of an RNA Sequencing Library

RNA sequencing was performed using corneal endothelial tissue from 60-week-old *Tcf4*^(CTG)100/(CTG)100^ (*n* = 5; five females) mice and wild-type controls (*n* = 5; two males and three females). After mouse euthanasia, the ocular globes were immediately preserved in RNAlater (Thermo Fisher Scientific, Waltham, MA, USA). The Descemet membrane with adherent corneal endothelium was carefully peeled from the corneal stroma and stored in RNAlater. Total RNA was isolated using the RNeasy Micro Kit (Qiagen, Hilden, Germany), with quality and quantity assessed using an Agilent 2100 Bioanalyzer with an RNA 6000 Pico Kit (Agilent Technologies, Santa Clara, CA, USA).

Libraries were prepared using the SMART-Seq HT Kit (Takara Bio Inc., Shiga, Japan) for full-length cDNA synthesis, followed by Illumina library construction using NexteraXT (Illumina Inc., San Diego, CA, USA). Sequencing was performed on the NovaSeq 6000 platform (Illumina Inc.) at the Genome Information Research Center, Osaka University. The RNA sequencing data have been deposited in the Gene Expression Omnibus database under accession number GSE283422.

### RNA Sequencing Data Analysis

The raw sequencing data quality was assessed using FastQC (v0.23.2; Babraham Bioinformatics; https://www.bioinformatics.babraham.ac.uk/projects/fastqc/) to evaluate read quality, GC-content, and sequence duplication levels. Reads were mapped to the mouse reference genome (GRCm39) using STAR (v2.7.11a),^35^ followed by gene expression quantification using RSEM (v1.3.1).^36^

Differential gene expression between *Tcf4*^(CTG)100/(CTG)100^ and wild-type mice was evaluated using DESeq2 (Bioconductor v3.14).^37^ Sex was included as a covariate in the DESeq2 design formula to account for potential sex-based variability between groups. Genes with *P* value < 0.05 and |Log_2_ Fold Change| ≥ 1.0 were considered to show significant differential expression. Gene expression patterns were visualized using volcano plots generated with ggplot2 (v3.5.1) in R (v4.1.3). Protein-coding differentially expressed genes (DEGs) were identified using biomaRt (v2.50.3)^38^ for subsequent analyses.

### Multivariate Analysis

Principal component analysis (PCA), heatmap analysis and correlation matrix analysis were performed using R. Expression values were normalized as log_10_ (1.0 + TPM), where TPM represents transcripts per million. PCA was conducted using the prcomp function. Hierarchical clustering was performed for heatmap analysis using the hclust function with the ward.D2 method, whereas correlation analysis was performed using Spearman's rank correlation (cor function) and visualized using corrplot for correlogram generation.

### Comparative Transcriptomic Analysis With Human FECD Data

To evaluate the translational relevance of our *Tcf4*^(CTG)100/(CTG)100^ mouse model, we performed a comparative analysis between mouse corneal endothelial transcriptome data and previously published RNA-Seq data from human FECD patients harboring *TCF4* trinucleotide repeat expansion (>50 CTG repeats).^39^^,^^40^ Differential gene expression data from both datasets were analyzed to identify overlapping genes using the VennDiagram package (v1.7.3) in R. Orthologous gene mapping between mouse and human genomes was performed using biomaRt. Shared dysregulated genes were identified based on consistent directional change (upregulation or downregulation) across both species. Venn diagrams were generated to visualize the overlap between the differentially expressed genes in both datasets.

### Pathway Analysis

Gene Ontology (GO) analysis was performed using ClusterProfiler (v4.2.2)^41^ and org.Mm.eg.db (v3.14.0). Enriched GO terms were identified using a *P* value threshold of 0.05 and categorized into biological process, cellular component, and molecular function. The top 10 terms from each category were visualized using ggplot2.

Pathway analysis was conducted using two approaches: Reactome pathway analysis using ReactomePA (v1.38.0) and Kyoto Encyclopedia of Genes and Genomes (KEGG) pathway analysis using ClusterProfiler. For both analyses, pathways with *P* value < 0.05 were considered significant. The top 10 enriched pathways were visualized using ggplot2, with plots showing gene ratios for each pathway.

### In Vivo Corneal Endothelial Assessment

Corneal endothelial imaging was performed on wild-type (*n* = 9; four males and five females) , *Tcf4*^+/(CTG)100^ mice (*n* = 10; six males and four females) and *Tcf4*^(CTG)100/(CTG)100^ mice (*n* = 12; five males and seven females) at 20, 40, and 60 weeks of age, corresponding to human age equivalents of approximately 15–20, 25–35, and 40–50 years, respectively.^42^ Mice were anesthetized using a Small Animal Anesthesia System (KN-1071-S; Natsume Seisakusho, Tokyo, Japan) with sevoflurane (Nikko Pharmaceutical, Tokyo, Japan) at 5% for induction and 2.5% to 4% for maintenance. After general anesthesia, topical anesthesia was administered using 2 µL of Benoxil ophthalmic solution (Santen Pharmaceutical, Osaka, Japan), corneal endothelial images were captured using a contact specular microscope (Cell Check C; Konan Medical, Hyogo, Japan). Although the initial experimental design aimed to extend observations to 80 to 100 weeks (equivalent to 60 to 80 human years),^42^ preliminary studies revealed increased mortality associated with general anesthesia and contact specular microscopy in older animals. Additionally, because characteristic symptoms were observed at 60 weeks, the observation period was limited to 60 weeks of age. Focused still images were extracted from video recordings following previously established methods.^43^ Comprehensive panoramic images were generated by merging 100 extracted images using AutoStitch64 software (originally developed at the University of British Columbia, Vancouver, BC, Canada).^44^ Guttae and cell borders were identified using a validated U-Net-based deep learning model.^43^ This model was previously developed by our group using manually annotated corneal endothelial images from *Col8a2*^L450W/L450W^ mice, where guttae and cell borders were labeled to train the U-Net architecture for automated detection. Using this established model, we calculated the percentage of guttae area and endothelial cell density (ECD) for the current study.

### Transmission Electron Microscopy Analysis

The ocular globes from *Tcf4*^(CTG)100/(CTG)100^ (*n* = 3; three females) and wild-type (*n* = 3; one male and two females) mice were fixed overnight at 4°C in 0.1M phosphate buffer (pH 7.4) containing 2% paraformaldehyde and 2% glutaraldehyde. The corneas were excised, washed in 0.1M phosphate buffer (4°C, 30 minutes), and post-fixed in 2% osmium tetroxide (4°C, three hours). After dehydration, specimens were embedded in epoxy resin, and ultrathin sections (70 nm) were collected on copper grids. Sections were counterstained with aqueous uranyl acetate and phosphotungstic acid (1 h each), followed by Reynolds' lead citrate (20 minutes). Additional staining was performed using 2% uranyl acetate (15 minutes) and lead stain solution (three minutes). Images were acquired using a JEM-1400Plus transmission electron microscope (JEOL, Tokyo, Japan) operating at 100 kV and equipped with a CCD camera.

## Results

### Generation of *Tcf4* Triplet–Repeat Knock-In Mice

To investigate the biological effects of TNR expansion in *Tcf4*, we used CRISPR/Cas9-mediated genome editing to generate knock-in mice harboring CTG repeats (*Tcf4*^+/(CTG)100^ and *Tcf4*^(CTG)100/(CTG)100^). A sequence containing 100 CTG repeats was specifically inserted into intron 2 of the murine *Tcf4* gene (Fig. 1A). After successful germline transmission and subsequent breeding, we confirmed the genotype of *Tcf4*^+/(CTG)100^ and *Tcf4*^(CTG)100/(CTG)100^ mice through genomic PCR analysis using specific primers (Supplementary Table S1) and Sanger sequencing validation. PCR genotyping analysis demonstrated the presence of a 1,144 bp amplicon in wild-type mice, both 1144 bp and 1456 bp amplicons in *Tcf4*^+/(CTG)100^ heterozygous mice, and only a 1456 bp amplicon in *Tcf4*^(CTG)100/(CTG)100^ homozygous mice, confirming the successful insertion of the CTG repeat sequence (Fig. 1B). The precise integration and sequence fidelity of the CTG repeat expansion were further verified through Sanger sequencing (Fig. 1C). Notably, the *Tcf4*^+/(CTG)100^ and *Tcf4*^(CTG)100/(CTG)100^ mice were viable and exhibited no overt phenotypic abnormalities under standard laboratory conditions. Additionally, since CTG repeat sequences have been reported to be unstable within the human genome in an age and cell type-specific context,^45^ we performed Sanger sequencing analysis using genomic DNA derived from tail tissues of *Tcf4*^(CTG)100/(CTG)100^ mice at 20 weeks (*n* = 2) and 60 weeks (*n* = 2) of age. Our results confirmed that all mice maintained CTG repeat sequences exceeding 95 repeats, indicating that the repeat length remained stable in mice during the observation period of this study (Supplementary Fig. S1).

### RNA-Seq of *Tcf4*^(CTG)100/(CTG)100^ and Wild-Type Mice

**Figure 2.**
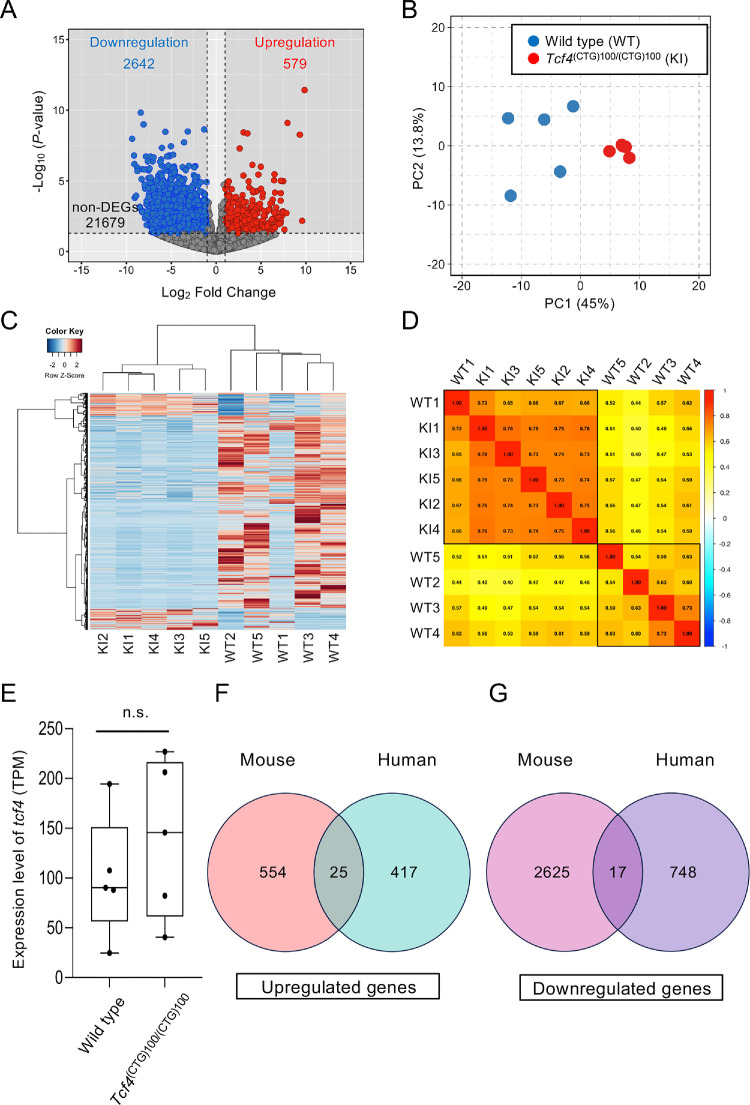
Transcriptome analysis of *Tcf4*^(CTG)100/(CTG)100^ mice corneal endothelium. **(A)** Volcano plot showing differentially expressed genes between *Tcf4*^(CTG)100/(CTG)100^ and wild-type mice. *Red* and *blue dots* represent significantly up-regulated (579 genes) and downregulated (2642 genes) genes, respectively (|Log_2_ Fold Change| ≥ 1 and *P* value < 0.05). *n* = 5 mice per group. **(B)** PCA plot demonstrating distinct clustering between *Tcf4*^(CTG)100/(CTG)100^ and wild-type samples. PC1 and PC2 account for 45.0% and 13.8% of the total variance, respectively. **(C)** Heatmap visualization with hierarchical clustering showing distinct gene expression patterns between the *Tcf4*^(CTG)100/(CTG)100^ and wild-type groups. *Red* indicates increased expression and *blue* indicates decreased expression. **(D)** Correlation matrix showing the relationships between individual samples. Higher correlation coefficients were observed within groups (0.44–0.73 for wild-type, 0.73–0.78 for *Tcf4*^(CTG)100/(CTG)100^) than between groups (0.40–0.72). **(E)** *Box plot* showing *Tcf4* gene expression levels (TPM) in wild-type and *Tcf4*^(CTG)100/(CTG)100^ corneal endothelial tissue. No significant difference in expression was observed between wild-type and *Tcf4*^(CTG)100/(CTG)100^ mice (*n* = 5 per group; *P* = 0.35). **(F)** Venn diagram showing the overlap of upregulated genes between human FECD patients with *TCF4* trinucleotide repeat expansion (>50 CTG repeats) (442 genes) and *Tcf4*^(CTG)100/(CTG)100^ mice (579 genes), with 25 shared genes identified. **(G)** Venn diagram depicting the overlap of downregulated genes between human FECD patients with *TCF4* trinucleotide repeat expansion (765 genes) and *Tcf4^(^*^CTG)100/(CTG)100^ mice (2642 genes), with 17 shared genes identified.

To investigate transcriptional changes associated with the CTG repeat expansion, we performed RNA sequencing analysis comparing *Tcf4*^(CTG)100/(CTG)100^ and wild-type mice. Among 24,900 expressed genes, we identified 3221 DEGs meeting our criteria of |Log₂ Fold Change| ≥ 1 and *P*-value < 0.05. Of these DEGs, 579 genes showed significant upregulation and 2642 genes showed significant downregulation in the *Tcf4*^(CTG)100/(CTG)100^ mice compared to the WT controls (Fig. 2A). PCA demonstrated a clear separation between *Tcf4*^(CTG)100/(CTG)100^ and WT samples along the first principal component, indicating distinct transcriptional profiles between the genotypes (Fig. 2B). We further characterized these differences by performing a hierarchical clustering analysis of the DEGs, which revealed distinct expression patterns between the *Tcf4*^(CTG)100/(CTG)100^ (*n* = 5) and wild-type (*n* = 5) groups (Fig. 2C). The resulting heatmap visualization showed clear segregation of samples by genotype, with upregulated genes depicted in red and downregulated genes in blue. We quantified the reproducibility of gene expression patterns within and between groups by calculating Pearson correlation coefficients for all sample pairs. The correlation analysis revealed high intragroup similarity, with correlation coefficients ranging from 0.44 to 0.73 among wild-type samples and 0.73 to 0.78 among *Tcf4*^(CTG)100/(CTG)100^ samples. By contrast, inter-group comparisons showed notably lower correlation coefficients (ranging from 0.40 to 0.72), further supporting the distinct transcriptional signatures between the genotypes, although one wild-type sample (WT1) showed higher correlation with *Tcf4*^(CTG)100/(CTG)100^ samples than with other wild-type samples (Fig. 2D). To investigate whether CTG repeat expansion affects *Tcf4* expression levels, we compared TPM values between wild-type and *Tcf4*^(CTG)100/(CTG)100^. Analysis of *Tcf4* expression levels showed no significant difference between *Tcf4*^(CTG)100/(CTG)100^ mice and wild-type mice (wild-type: 101.01 ± 61.06 TPM vs. *Tcf4*^(CTG)100/(CTG)100^: 140.32 ± 79.40 TPM; *P* = 0.35) (Fig. 2E). To determine the translational relevance of our mouse model, we assessed the overlap between DEGs identified in *Tcf4*^(CTG)100/(CTG)100^ mice and those previously reported in human FECD patients with *TCF4* trinucleotide repeat expansion (>50 CTG repeats). Venn diagram analysis revealed 25 shared upregulated genes between mouse (579 genes) and human (442 genes) datasets (Fig. 2F). Similarly, 17 shared downregulated genes were identified between mouse (2642 genes) and human (765 genes) datasets (Fig. 2G).

### GO and Pathway Enrichment Analysis

**Figure 3.**
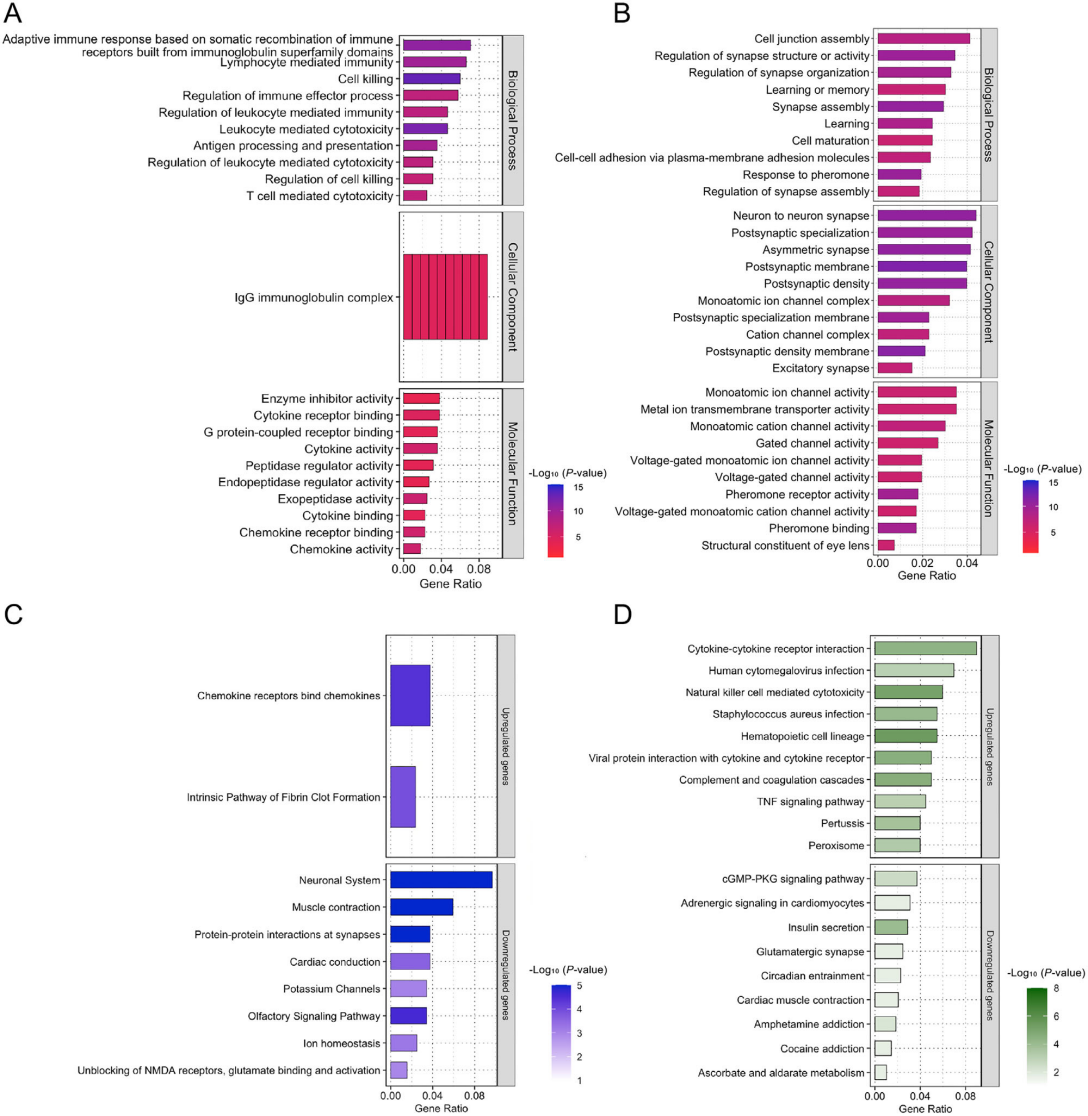
GO and pathway enrichment analysis of differentially expressed genes in *Tcf4*^(CTG)100/(CTG)100^ mice. **(A)** GO analysis of upregulated genes showing enrichment in biological processes, cellular components, and molecular functions. The top enriched terms included adaptive immune response based on somatic recombination of immune receptors, lymphocyte mediated immunity, and regulation of immune effector process. Bar colors indicate −log_10_ (*P* value). *n* = 5 mice per group. **(B)** GO analysis of downregulated genes revealing enrichment in cell junction assembly, regulation of synapse structure or activity, and regulation of synapse organization pathways. Neuron to neuron synapse, postsynaptic specialization, and asymmetric synapse were among the enriched cellular components. **(C)** Reactome pathway analysis showing significant enrichment of up-regulated genes in chemokine receptors bind chemokines and intrinsic pathway of fibrin clot formation pathways. Downregulated genes were enriched in neuronal system, muscle contraction, and protein-protein interactions at synapses. **(D)** KEGG pathway analysis highlighting enrichment of up-regulated genes in cytokine-cytokine receptor interaction, human cytomegalovirus infection, natural killer cell-mediated cytotoxicity, and *Staphylococcus aureus* infection pathways. Downregulated genes were primarily associated with cGMP-PKG signaling pathway, adrenergic signaling in cardiomyocytes, and insulin secretion.

GO enrichment analysis revealed significant enrichment of 181 biological processes, 72 cellular components, and 49 molecular function terms among the DEGs (*P* < 0.05). The top 10 significantly enriched terms for each GO category are presented in Figure 3.

Among the upregulated genes, the enriched biological process terms included adaptive immune response based on somatic recombination of immune receptors built from immunoglobulin superfamily domains, lymphocyte mediated immunity, and regulation of immune effector process. The enriched cellular component terms were dominated by IgG immunoglobulin complex. Molecular function analysis revealed enrichment in enzyme inhibitor activity, cytokine receptor binding, and G protein-coupled receptor binding (Fig. 3A). By contrast, downregulated genes showed significant enrichment in biological process terms related to cell junction assembly, regulation of synapse structure or activity, and regulation of synapse organization. Cellular component analysis of downregulated genes identified enrichment in neuron to neuron synapse, postsynaptic specialization, and asymmetric synapse. Molecular function terms included monoatomic ion channel activity, metal ion transmembrane transporter activity, and monoatomic cation channel activity (Fig. 3B).

Pathway enrichment analysis using Reactome revealed a significant association between upregulated genes and chemokine receptors bind chemokines and intrinsic pathway of fibrin clot formation. Downregulated genes showed enrichment in neuronal system, muscle contraction, and protein-protein interactions at synapses (Fig. 3C). KEGG pathway analysis of upregulated genes identified significant enrichment in cytokine–cytokine receptor interaction, human cytomegalovirus infection, natural killer cell-mediated cytotoxicity, and *Staphylococcus aureus* infection pathways. Downregulated genes were primarily associated with cGMP-PKG signaling pathway, adrenergic signaling in cardiomyocytes, and insulin secretion (Fig. 3D).

### Analysis of the Formation of Guttae and Cell Density

**Figure 4.**
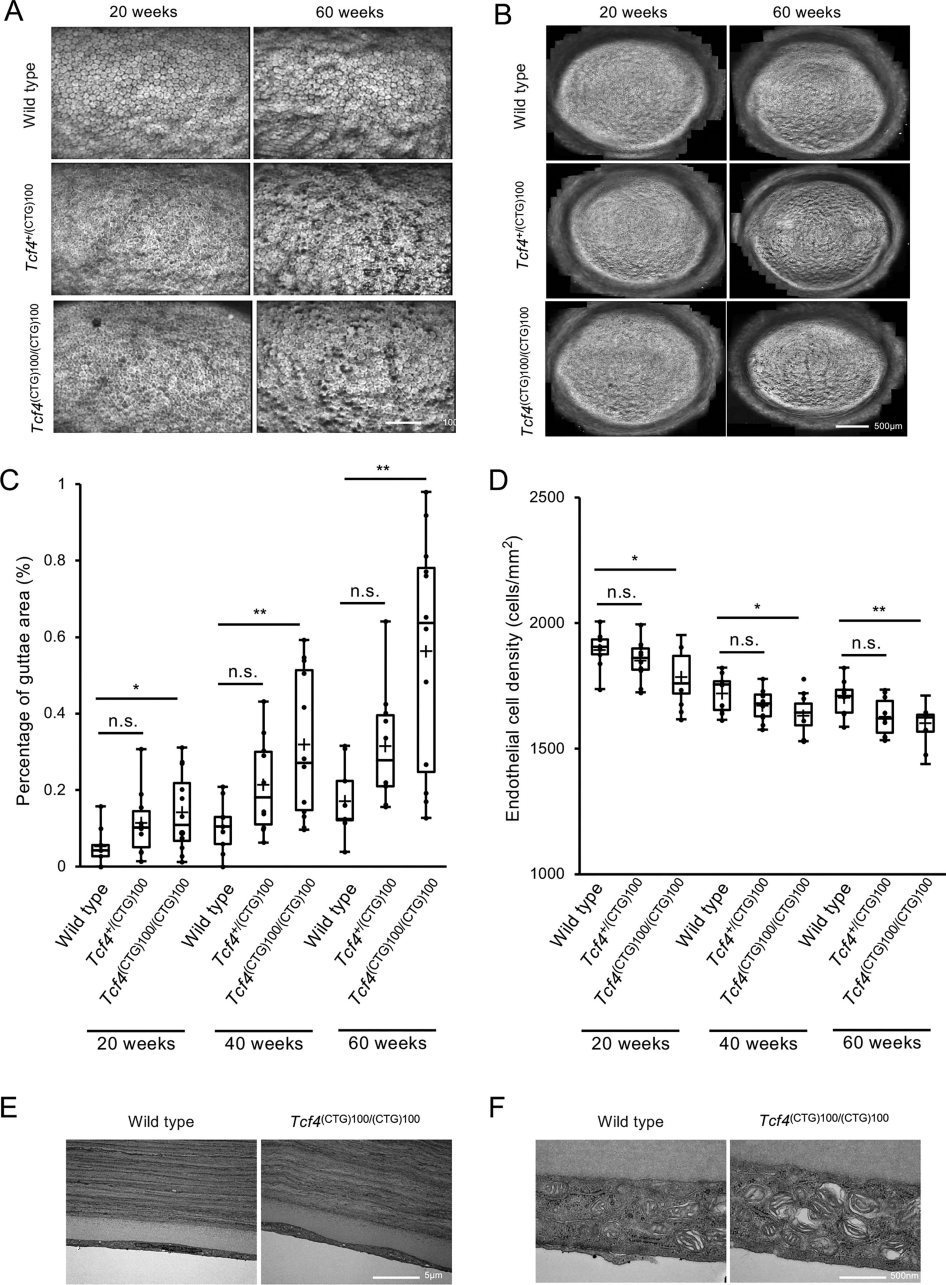
In vivo corneal endothelium assessment of *Tcf4*^+/(CTG)100^ and *Tcf4*^(CTG)100/(CTG)100^ mice. **(A)** Representative specular microscopy images of corneal endothelium at 20 and 60 weeks of age, showing that *Tcf4*^+/(CTG)100^ and *Tcf4*^(CTG)100/(CTG)100^ mice have increased guttae formation and altered cell morphology compared to wild-type mice. *Scale bar*: 100 µm. **(B)** Panoramic images of corneal endothelium demonstrating the progression of endothelial changes over time in *Tcf4*^+/(CTG)100^, *Tcf4*^(CTG)100/(CTG)100^ and wild-type mice. *Scale bar*: 500 µm. **(C)** Quantitative analysis of guttae formation in *Tcf4*^+/(CTG)100^ (*n* = 10), *Tcf4*^(CTG)100/(CTG)100^ (*n* = 12) and wild-type mice (*n* = 9). One image per mouse was analyzed, with each *dot* representing a single data point from an individual mouse. Heterozygous *Tcf4*^+/(CTG)100^ mice demonstrated progressive guttae formation compared to wild-type controls, although differences were not statistically significant (20 weeks: 0.114% ± 0.082% vs. 0.053% ± 0.047%, *P* = 0.220; 40 weeks: 0.214% ± 0.118% vs. 0.105% ± 0.065%, *P* = 0.184; 60 weeks: 0.314% ± 0.145% vs. 0.170% ± 0.089%, *P* = 0.264). Guttae area percentage progressively increased with age in *Tcf4*^(CTG)100/(CTG)100^ mice compared to wild-type controls (20 weeks: 0.141% ± 0.099% vs. 0.053% ± 0.047%, *P* = 0.050; 40 weeks: 0.319% ± 0.181% vs. 0.105% ± 0.065%, *P* = 0.003; 60 weeks: 0.563% ± 0.293% vs. 0.170% ± 0.089%, *P* < 0.001). **(D)** Analysis of endothelial cell density in *Tcf4*^+/(CTG)100^ (*n* = 10), *Tcf4*^(CTG)100/(CTG)100^ (*n* = 12) and wild-type mice (*n* = 9). Heterozygous *Tcf4*^+/(CTG)100^ mice showed a trend toward reduced ECD values compared to wild-type controls, although these differences did not reach statistical significance despite the consistent pattern of decline (20 weeks: 1852 ± 78 vs. 1895 ± 71 cells/mm², *P* = 0.508; 40 weeks: 1673 ± 61 vs. 1,721 ± 72 cells/mm², *P* = 0.236; 60 weeks: 1629 ± 71 vs. 1,704 ± 68 cells/mm², *P* = 0.070). In contrast, significant reduction in ECD was observed in *Tcf4*^(CTG)100/(CTG)100^ mice compared to wild-type controls at all time points (20 weeks: 1786 ± 108 vs. 1895 ± 71 cells/mm^2^, *P* = 0.025; 40 weeks: 1632 ± 66 vs. 1721 ± 72 cells/mm^2^, *P* = 0.013; 60 weeks: 1600 ± 76 vs. 1704 ± 68 cells/mm^2^, *P* = 0.008). **(E)** Transmission electron microscopy analysis of Descemet's membrane in 60-week-old mice. *Tcf4*^(CTG)100/(CTG)100^ mice exhibited distinctive undulating patterns with focal thickening compared to wild-type controls. *Scale bar*: 5 µm. **(F)** Ultrastructural analysis at higher magnification demonstrated enlarged endoplasmic reticulum and distended mitochondria in corneal endothelial cells of *Tcf4*^(CTG)100/(CTG)100^ mice, while corneal endothelial cells from wild-type mice exhibited normal organelle architecture. *Scale bar*: 500 nm.

Corneal endothelial morphology was evaluated in *Tcf4*^+/(CTG)100^ (*n* = 10), *Tcf4*^(CTG)100/(CTG)100^ (*n* = 12), and wild-type (*n* = 9) mice using contact specular microscopy. Representative specular microscopy images at 20 and 60 weeks of age demonstrated a more extensive development of guttae, enlargement of corneal endothelial cells, and reduction in endothelial cell density in both *Tcf4*^+/(CTG)100^ and *Tcf4*^(CTG)100/(CTG)100^ mice compared to wild-type controls (Fig. 4A). Panoramic imaging confirmed the presence of guttae specifically in the *Tcf4*^+/(CTG)100^ and *Tcf4*^(CTG)100/(CTG)100^ mice, whereas wild-type mice showed no obvious guttae formation (Fig. 4B). Heterozygous *Tcf4*^+/(CTG)100^ mice showed a trend toward increased guttae formation compared to wild-type controls, though these differences did not reach statistical significance at any time point (20 weeks: 0.114% ± 0.082% vs. 0.053% ± 0.047%, *P* = 0.220; 40 weeks: 0.214% ± 0.118% vs. 0.105% ± 0.065%, *P* = 0.184; 60 weeks: 0.314% ± 0.145% vs. 0.170% ± 0.089%, *P* = 0.264). In contrast, homozygous *Tcf4*^(CTG)100/(CTG)100^ mice exhibited significantly higher guttae area compared to wild-type controls at all time points (20 weeks: 0.141% ± 0.099% vs. 0.053% ± 0.047%, *P* = 0.050; 40 weeks: 0.319% ± 0.181% vs. 0.105% ± 0.065%, *P* = 0.003; 60 weeks: 0.563% ± 0.293% vs. 0.170% ± 0.089%, *P* < 0.001) (Fig. 4C).

ECD analysis revealed a similar pattern, with heterozygous *Tcf4*^+/(CTG)100^ mice showing a numerical decrease in endothelial cell density compared to wild-type controls, particularly at 60 weeks, although these differences did not achieve statistical significance (20 weeks: 1852 ± 78 vs. 1895 ± 71 cells/mm^2^, *P* = 0.508; 40 weeks: 1673 ± 61 vs. 1,721 ± 72 cells/mm^2^, *P* = 0.236; 60 weeks: 1629 ± 71 vs. 1704 ± 68 cells/mm^2^, *P* = 0.070). In homozygous *Tcf4*^(CTG)100/(CTG)100^ mice, a statistically significant reduction in ECD was observed compared to wild-type controls at all time points (20 weeks: 1786 ± 108 vs. 1895 ± 71 cells/mm^2^, *P* = 0.025; 40 weeks: 1632 ± 66 vs. 1721 ± 72 cells/mm^2^, *P* = 0.013; 60 weeks: 1600 ± 76 vs. 1704 ± 68 cells/mm^2^, *P* = 0.008) (Fig. 4D). These findings demonstrate progressive corneal endothelial changes that parallel features of human FECD, with a more pronounced and statistically significant phenotype in homozygous mice, and a milder, dose-dependent effect in heterozygous mice that did not reach statistical significance.

Transmission electron microscope analysis performed on corneal tissues from 60-week-old *Tcf4*^(CTG)100/(CTG)100^ and wild-type mice revealed a smooth and uniform Descemet membrane without apparent irregularities in the wild-type mice, whereas the *Tcf4*^(CTG)100/(CTG)100^ mice demonstrated distinctive undulating patterns with focal thickening of the Descemet membrane, corresponding to the guttae formations previously observed by specular microscopy (Fig. 4E). Higher magnification examination revealed dilated endoplasmic reticulum and swollen mitochondria in the corneal endothelial cells of *Tcf4*^(CTG)100/(CTG)100^ mice, whereas wild-type corneal endothelial cells maintained the normal morphology of both organelles (Fig. 4F).

## Discussion

In the present study, we generated a knock-in mouse model carrying 100 CTG TNR in the intronic region of *Tcf4*. Although the TNR was located in a noncoding region and thus not directly encoding any protein products, RNA sequencing analysis revealed widespread dysregulation of gene expression. Most notably, this knock-in mouse model exhibited two hallmark features characteristic of human FECD: the formation of guttae and decreased corneal endothelial cell density.^1^^–^^3^ Furthermore, we observed evidence suggestive of endoplasmic reticulum stress and associated mitochondrial dysfunction, paralleling the pathological findings in human FECD.^46^^–^^50^ These findings strongly suggest that our mouse model recapitulates key aspects of human FECD pathogenesis.

The discovery of an expanded TNR in *TCF4* as a predominant genetic feature in FECD^13^ has stimulated extensive research into its pathogenic mechanisms.^22^^–^^29^ Multiple molecular pathways have been proposed to explain FECD pathogenesis associated with *TCF4* TNR expansion. One potential mechanism involves altered *TCF4* expression.^24^^,^^28^^,^^29^ A second mechanism centers on RNA toxicity, which is well established in the pathogenesis of myotonic dystrophy types 1 and 2.^51^^,^^52^ This hypothesis is supported by consistent observations of MBNL sequestration and aberrant splicing patterns in FECD corneal tissue.^22^^,^^23^ Additionally, repeat-associated non-AUG translation may generate toxic peptides that compromise cellular function.^25^ The documented somatic instability of the CTG18.1 repeat may increase with age and contribute to disease progression, particularly given that corneal endothelial cells are post-mitotic and continuously exposed to environmental stressors.^26^^,^^27^^,^^45^ However, these proposed mechanisms primarily address corneal endothelial cell dysfunction, rather than explaining how TNR expansion leads to guttae formation, the hallmark feature of FECD.

Because of the complex interactions between genetic and environmental factors in human FECD,^3^ isolating the specific effects of TNR expansion has been challenging. Our mouse model demonstrates that TNR expansion on its own is sufficient to induce guttae formation in vivo, thereby providing crucial evidence for understanding the fundamental mechanisms of FECD pathogenesis. Gene regulation involves complex mechanisms that extend beyond protein-coding sequences, as the noncoding regions of the genome significantly influence the expression of multiple genes through various mechanisms. These include enhancer and silencer elements that modulate transcription, structural elements that affect chromatin organization, and non-coding RNAs that regulate post-transcriptional processes.^53^^–^^55^

In this study, we observed a notable gene dosage effect, with homozygous *Tcf4*^(CTG)100/(CTG)100^ mice showing significantly greater guttae formation and ECD reduction compared to wild-type controls at all time points. Heterozygous *Tcf4*^+/(CTG)100^ mice displayed an intermediate phenotype, trending toward increased pathology versus wild-type, though not reaching statistical significance. This pattern suggests CTG18.1 expansion confers gain-of-function effects through mechanisms like RNA toxicity, with pathology severity corresponding to CTG repeat dosage. This gene dosage effect provides important mechanistic insights difficult to establish in human FECD patients because of confounding factors including diverse genetic backgrounds, environmental influences, and variations in disease progression. Homozygous CTG18.1 expansion carriers are also rare in human populations, complicating direct comparisons while controlling for relevant variables. Our mouse model isolates the specific effects of CTG repeat dosage on corneal endothelial pathology. The observed dose-dependent relationship between CTG expansion and disease severity supports gain-of-function mechanisms in FECD pathogenesis and suggests therapeutic strategies targeting RNA toxicity might be beneficial. Further analysis of this model may provide deeper insights into how *TCF4* repeat expansion contributes to FECD pathogenesis.

Currently, corneal transplantation using donor tissue remains the primary treatment for FECD.^1^^–^^3^ However, nonsurgical therapeutic approaches, including antisense oligonucleotides and CRISPR/Cas9-based strategies targeting TNR expansion, have been proposed using cell models carrying repeat expansions.^18^^,^^56^ While existing FECD mouse models based on *Col8a2* mutations (modeling early-onset FECD)^57^ and UV-induced endothelial cell death^58^ have proven valuable, a TNR expansion model is essential for developing therapies that specifically target the most common form of human FECD. The mouse model established in this study therefore represents a crucial tool for accelerating the development of both pharmacological and genetic therapeutic strategies, particularly those targeting TNR-mediated pathogenesis.

The limitation of the present study is that it does not precisely define the mechanisms by which TNR expansion induces guttae formation and cellular dysfunction. Further investigation is needed to determine whether the pathogenic mechanisms previously reported in human FECD are also present in this mouse model. Our transcriptomic analysis revealed a limited overlap of differentially expressed genes between *Tcf4*^(CTG)100/(CTG)100^ mice and human FECD patients with *TCF4* repeat expansion—specifically, 25 shared upregulated and 17 shared downregulated genes. Although these represent a small fraction of total DEGs in each species, these conserved changes may highlight important pathways in TNR expansion-mediated pathology. However, we acknowledge that most gene expression changes were species-specific, and the significance of this overlap requires further evaluation. These conserved DEGs warrant focused investigation to determine their contributions to FECD pathogenesis. In our knock-in mouse model, subtle guttae formation was observed at 20 weeks (equivalent to human age 10–20 years), with FECD phenotype manifestation by 60 weeks (equivalent to human age 40–50 years). While this timing appears comparable to typical human FECD, which is commonly diagnosed between 40–50 years of age, the disease onset in our model may be slightly earlier than in human cases. Important species differences must also be considered when using this mouse model for future research. Unlike human corneal endothelium, mouse corneal endothelial cells retain some regenerative capacity. Although our model developed the FECD phenotype despite this potential regenerative ability, it remains unclear whether disease progression mirrors that of human FECD or whether it ultimately leads to bullous keratopathy as observed in humans. Although extended observation beyond 60 weeks was precluded by increased mortality risk associated with general anesthesia and examination procedures, longitudinal studies of phenotype progression would be valuable for several reasons. Such studies would help evaluate the translational relevance of this mouse model to human disease, enable detailed characterization of stage-specific pathological changes, and assess therapeutic responses across different disease stages.

In conclusion, we have demonstrated that TNR expansion in the *Tcf4* intron, on its own and independent of environmental factors, is sufficient to induce the FECD phenotype. This mouse model represents a valuable tool that will accelerate both the elucidation of disease mechanisms and the development of novel therapeutic strategies for FECD.

## Supplementary Material

Supplement 1

Supplement 2

## References

[1] Eghrari AO, Riazuddin SA, Gottsch JD. Fuchs corneal dystrophy. *Prog Mol Biol Transl Sci*. 2015; 134: 79–97. 26310151 10.1016/bs.pmbts.2015.04.005

[2] Fautsch MP, Wieben ED, Baratz KH, et al. TCF4-mediated Fuchs endothelial corneal dystrophy: insights into a common trinucleotide repeat-associated disease. *Prog Retin Eye Res*. 2021; 81: 100883. 32735996 10.1016/j.preteyeres.2020.100883 PMC7988464

[3] Ong Tone S, Kocaba V, Bohm M, Wylegala A, White TL, Jurkunas UV. Fuchs endothelial corneal dystrophy: the vicious cycle of Fuchs pathogenesis. *Prog Retin Eye Res*. 2021; 80: 100863. 32438095 10.1016/j.preteyeres.2020.100863 PMC7648733

[4] Aiello F, Gallo Afflitto G, Ceccarelli F, Cesareo M, Nucci C. Global prevalence of fuchs endothelial corneal dystrophy (FECD) in adult population: a systematic review and meta-analysis. *J Ophthalmol*. 2022; 2022(1): 3091695. 35462618 10.1155/2022/3091695 PMC9023201

[5] Bourne WM, Johnson DH, Campbell RJ. The ultrastructure of Descemet's membrane. III. Fuchs' dystrophy. *Arch Ophthalmol*. 1982; 100: 1952–1955. 6983339 10.1001/archopht.1982.01030040932013

[6] Louttit MD, Kopplin LJ, Igo RP Jr., et al. A multicenter study to map genes for Fuchs endothelial corneal dystrophy: baseline characteristics and heritability. *Cornea*. 2012; 31: 26–35. 22045388 10.1097/ICO.0b013e31821c9b8f PMC3719980

[7] Zhang J, Patel DV. The pathophysiology of Fuchs' endothelial dystrophy—a review of molecular and cellular insights. *Exp Eye Res*. 2015; 130: 97–105. 25446318 10.1016/j.exer.2014.10.023

[8] Watanabe S, Oie Y, Fujimoto H, et al. Relationship between corneal guttae and quality of vision in patients with mild Fuchs’ endothelial corneal dystrophy. *Ophthalmology*. 2015; 122: 2103–2109. 26189189 10.1016/j.ophtha.2015.06.019

[9] Wacker K, McLaren JW, Amin SR, Baratz KH, Patel SV. Corneal high-order aberrations and backscatter in Fuchs’ endothelial corneal dystrophy. *Ophthalmology*. 2015; 122: 1645–1652. 26050543 10.1016/j.ophtha.2015.05.005 PMC4516693

[10] Lass JH, Sugar A, Benetz BA, et al. Endothelial cell density to predict endothelial graft failure after penetrating keratoplasty. *Arch Ophthalmol*. 2010; 128: 63–69. 20065219 10.1001/archophthalmol.2010.128.63 PMC2950322

[11] Vaiciuliene R, Rylskyte N, Baguzyte G, Jasinskas V. Risk factors for fluctuations in corneal endothelial cell density (Review). *Exp Ther Med*. 2022; 23: 129. 34970352 10.3892/etm.2021.11052 PMC8713183

[12] Krachmer JH, Purcell JJ Jr., Young CW, Bucher KD. Corneal endothelial dystrophy. A study of 64 families. *Arch Ophthalmol*. 1978; 96: 2036–2039. 309758 10.1001/archopht.1978.03910060424004

[13] Wieben ED, Aleff RA, Tosakulwong N, et al. A common trinucleotide repeat expansion within the transcription factor 4 (TCF4, E2-2) gene predicts Fuchs corneal dystrophy. *PloS one*. 2012; 7: e49083. 23185296 10.1371/journal.pone.0049083 PMC3504061

[14] Mootha VV, Gong X, Ku HC, Xing C. Association and familial segregation of CTG18.1 trinucleotide repeat expansion of TCF4 gene in Fuchs' endothelial corneal dystrophy. *Invest Ophthalmol Vis Sci*. 2014; 55: 33–42. 24255041 10.1167/iovs.13-12611 PMC3880006

[15] Xing C, Gong X, Hussain I, et al. Transethnic replication of association of CTG18.1 repeat expansion of TCF4 gene with Fuchs' corneal dystrophy in Chinese implies common causal variant. *Invest Ophthalmol Vis Sci*. 2014; 55: 7073–7078. 25298419 10.1167/iovs.14-15390 PMC4224583

[16] Nakano M, Okumura N, Nakagawa H, et al. Trinucleotide repeat expansion in the TCF4 gene in Fuchs’ endothelial corneal dystrophy in Japanese. *Invest Ophthalmol Vis Sci*. 2015; 56: 4865–4869. 26218914 10.1167/iovs.15-17082

[17] Rao BS, Tharigopala A, Rachapalli SR, Rajagopal R, Soumittra N. Association of polymorphisms in the intron of TCF4 gene to late-onset Fuchs endothelial corneal dystrophy: an Indian cohort study. *Indian J Ophthalmol*. 2017; 65: 931–935. 29044056 10.4103/ijo.IJO_191_17 PMC5678327

[18] Zarouchlioti C, Sanchez-Pintado B, Tear NJH, et al. Antisense therapy for a common corneal dystrophy ameliorates TCF4 repeat expansion-mediated toxicity. *The Am J Hum Genet*. 2018; 102: 528–539. 29526280 10.1016/j.ajhg.2018.02.010 PMC5985359

[19] Okumura N, Hayashi R, Nakano M, et al. Association of rs613872 and trinucleotide repeat expansion in the TCF4 gene of German patients with Fuchs endothelial corneal dystrophy. *Cornea*. 2019; 38: 799–805. 30973406 10.1097/ICO.0000000000001952 PMC6554040

[20] Okumura N, Puangsricharern V, Jindasak R, et al. Trinucleotide repeat expansion in the transcription factor 4 (TCF4) gene in Thai patients with Fuchs endothelial corneal dystrophy. *Eye (Lond)*. 2020; 34: 880–885. 31554942 10.1038/s41433-019-0595-8 PMC7182560

[21] Viberg A, Westin IM, Golovleva I, Byström B. TCF4 trinucleotide repeat expansion in Swedish cases with Fuchs' endothelial corneal dystrophy. *Acta Ophthalmol*. 2022; 100: 541–548. 34644448 10.1111/aos.15032

[22] Du J, Aleff RA, Soragni E, et al. RNA toxicity and missplicing in the common eye disease Fuchs endothelial corneal dystrophy. *J Biol Chem*. 2015; 290: 5979–5990. 25593321 10.1074/jbc.M114.621607 PMC4358235

[23] Mootha VV, Hussain I, Cunnusamy K, et al. TCF4 Triplet repeat expansion and nuclear RNA foci in Fuchs’ endothelial corneal dystrophy. *Invest Ophthalmol Vis Sci*. 2015; 56: 2003–2011. 25722209 10.1167/iovs.14-16222 PMC4373545

[24] Foja S, Luther M, Hoffmann K, Rupprecht A, Gruenauer-Kloevekorn C. CTG18.1 repeat expansion may reduce TCF4 gene expression in corneal endothelial cells of German patients with Fuchs' dystrophy. *Graefes Arch Clin Exp Ophthalmol*. 2017. 10.1007/s00417-017-3697-7 28608272

[25] Soragni E, Petrosyan L, Rinkoski TA, et al. Repeat-associated non-ATG (RAN) translation in Fuchs’ endothelial corneal dystrophy. *Invest Ophthalmol Vis Sci*. 2018; 59: 1888–1896. 29677349 10.1167/iovs.17-23265 PMC5886103

[26] Soh YQ, Peh Swee Lim G, Htoon HM, et al. Trinucleotide repeat expansion length as a predictor of the clinical progression of Fuchs’ endothelial corneal dystrophy. *PloS One*. 2019; 14: e0210996. 30682148 10.1371/journal.pone.0210996 PMC6347165

[27] Hafford-Tear NJ, Tsai YC, Sadan AN, et al. CRISPR/Cas9-targeted enrichment and long-read sequencing of the Fuchs endothelial corneal dystrophy-associated TCF4 triplet repeat. *Genet Med*. 2019; 21: 2092–2102. 30733599 10.1038/s41436-019-0453-x PMC6752322

[28] Okumura N, Hayashi R, Nakano M, et al. Effect of trinucleotide repeat expansion on the expression of TCF4 mRNA in Fuchs’ endothelial corneal dystrophy. *Invest Ophthalmol Vis Sci*. 2019; 60: 779–786. 30811544 10.1167/iovs.18-25760 PMC6392475

[29] Honda T, Nakagawa T, Yuasa T, et al. Dysregulation of the TCF4 isoform in corneal endothelial cells of patients with Fuchs endothelial corneal dystrophy. *Invest Ophthalmol Vis Sci*. 2024; 65: 27. 10.1167/iovs.65.6.27 PMC11185267 38884552

[30] Naito Y, Hino K, Bono H, Ui-Tei K. CRISPRdirect: software for designing CRISPR/Cas guide RNA with reduced off-target sites. *Bioinformatics*. 2015; 31: 1120–1123. 25414360 10.1093/bioinformatics/btu743 PMC4382898

[31] Mashiko D, Fujihara Y, Satouh Y, Miyata H, Isotani A, Ikawa M. Generation of mutant mice by pronuclear injection of circular plasmid expressing Cas9 and single guided RNA. *Sci Rep*. 2013; 3: 3355. 24284873 10.1038/srep03355 PMC3842082

[32] Fujihara Y, Ikawa M. CRISPR/Cas9-based genome editing in mice by single plasmid injection. *Methods Enzymol*. 2014; 546: 319–336. 25398347 10.1016/B978-0-12-801185-0.00015-5

[33] Fujihara Y, Kaseda K, Inoue N, Ikawa M, Okabe M. Production of mouse pups from germline transmission-failed knockout chimeras. *Transgenic Res*. 2013; 22: 195–200. 22826106 10.1007/s11248-012-9635-x

[34] Oji A, Noda T, Fujihara Y, et al. CRISPR/Cas9 mediated genome editing in ES cells and its application for chimeric analysis in mice. *Sci Rep*. 2016; 6: 31666. 27530713 10.1038/srep31666 PMC4987700

[35] Dobin A, Davis CA, Schlesinger F, et al. STAR: ultrafast universal RNA-seq aligner. *Bioinformatics*. 2013; 29: 15–21. 23104886 10.1093/bioinformatics/bts635 PMC3530905

[36] Li B, Dewey CN. RSEM: accurate transcript quantification from RNA-Seq data with or without a reference genome. *BMC Bioinformatics*. 2011; 12: 323. 21816040 10.1186/1471-2105-12-323 PMC3163565

[37] Love MI, Huber W, Anders S. Moderated estimation of fold change and dispersion for RNA-seq data with DESeq2. *Genome Biol*. 2014; 15: 550. 25516281 10.1186/s13059-014-0550-8 PMC4302049

[38] Durinck S, Spellman PT, Birney E, Huber W. Mapping identifiers for the integration of genomic datasets with the R/Bioconductor package biomaRt. *Nat Protoc*. 2009; 4: 1184–1191. 19617889 10.1038/nprot.2009.97 PMC3159387

[39] Tokuda Y, Okumura N, Komori Y, et al. Transcriptome dataset of human corneal endothelium based on ribosomal RNA-depleted RNA-Seq data. *Sci Data*. 2020; 7: 407. 33219220 10.1038/s41597-020-00754-1 PMC7680133

[40] Nakagawa T, Tokuda Y, Nakano M, et al. RNA-Seq–based transcriptome analysis of corneal endothelial cells derived from patients with Fuchs endothelial corneal dystrophy. *Sci Rep*. 2023; 13: 8647. 37244951 10.1038/s41598-023-35468-y PMC10224979

[41] Wu T, Hu E, Xu S, et al. ClusterProfiler 4.0: a universal enrichment tool for interpreting omics data. *Innovation (Camb)*. 2021; 2: 100141. 34557778 10.1016/j.xinn.2021.100141 PMC8454663

[42] Dutta S, Sengupta P. Men and mice: relating their ages. *Life Sci*. 2016; 152: 244–248. 26596563 10.1016/j.lfs.2015.10.025

[43] Okumura N, Yamada S, Nishikawa T, et al. U-Net convolutional neural network for segmenting the corneal endothelium in a mouse model of Fuchs endothelial corneal dystrophy. *Cornea*. 2022; 41: 901–907. 34864800 10.1097/ICO.0000000000002956

[44] Brown M, Lowe DG. Automatic panoramic image stitching using invariant features. *Int J Comput Vis*. 2007; 74: 59–73.

[45] Zarouchlioti C, Efthymiou S, Facchini S, et al. Tissue-specific TCF4 triplet repeat instability revealed by optical genome mapping. *EBioMedicine*. 2024; 108: 105328. 39278108 10.1016/j.ebiom.2024.105328 PMC11419830

[46] Engler C, Kelliher C, Spitze AR, Speck CL, Eberhart CG, Jun AS. Unfolded protein response in fuchs endothelial corneal dystrophy: a unifying pathogenic pathway? *Am J Ophthalmol*. 2010; 149: 194–202.e192. 20103053 10.1016/j.ajo.2009.09.009 PMC2813215

[47] Okumura N, Kitahara M, Okuda H, et al. Sustained activation of the unfolded protein response induces cell death in Fuchs’ endothelial corneal dystrophy. *Invest Ophthalmol Vis Sci*. 2017; 58: 3697–3707. 28727885 10.1167/iovs.16-21023

[48] Okumura N, Hashimoto K, Kitahara M, et al. Activation of TGF-β signaling induces cell death via the unfolded protein response in Fuchs endothelial corneal dystrophy. *Sci Rep*. 2017; 7: 6801. 28754918 10.1038/s41598-017-06924-3 PMC5533742

[49] Kumar V, Jurkunas UV. Mitochondrial dysfunction and mitophagy in Fuchs endothelial corneal dystrophy. *Cells*. 2021; 10: 1888. 34440658 10.3390/cells10081888 PMC8392447

[50] Qureshi S, Lee S, Steidl W, et al. Endoplasmic reticulum stress disrupts mitochondrial bioenergetics, dynamics and causes corneal endothelial cell apoptosis. *Invest Ophthalmol Vis Sci*. 2023; 64: 18. 10.1167/iovs.64.14.18 PMC10653263 37962528

[51] Braz SO, Acquaire J, Gourdon G, Gomes-Pereira M. Of mice and men: advances in the understanding of neuromuscular aspects of myotonic dystrophy. *Front Neurol*. 2018; 9: 519. 30050493 10.3389/fneur.2018.00519 PMC6050950

[52] Sznajder ŁJ, Swanson MS. Short tandem repeat expansions and RNA-mediated pathogenesis in myotonic dystrophy. *Int J Mol Sci*. 2019; 20: 3365. 31323950 10.3390/ijms20133365 PMC6651174

[53] Epstein DJ. Cis-regulatory mutations in human disease. *Brief Funct Genomics Proteomics*. 2009; 8: 310–316. 10.1093/bfgp/elp021 PMC2742803 19641089

[54] Spielmann M, Klopocki E. CNVs of noncoding cis-regulatory elements in human disease. *Curr Opin Genet Dev*. 2013; 23: 249–256. 23601627 10.1016/j.gde.2013.02.013

[55] Zhang F, Lupski JR. Non-coding genetic variants in human disease. *Hum Mol Genet*. 2015; 24: R102–R110. 26152199 10.1093/hmg/ddv259 PMC4572001

[56] Rong Z, Gong X, Hulleman JD, Corey DR, Mootha VV. Trinucleotide repeat-targeting dCas9 as a therapeutic strategy for Fuchs’ endothelial corneal dystrophy. *Transl Vis Sci Technol*. 2020; 9: 47. 10.1167/tvst.9.9.47 PMC7463221 32934897

[57] Jun AS, Meng H, Ramanan N, et al. An alpha 2 collagen VIII transgenic knock-in mouse model of Fuchs endothelial corneal dystrophy shows early endothelial cell unfolded protein response and apoptosis. *Hum Mol Genet*. 2012; 21: 384–393. 22002996 10.1093/hmg/ddr473 PMC3276279

[58] Liu C, Miyajima T, Melangath G, et al. Ultraviolet A light induces DNA damage and estrogen-DNA adducts in Fuchs endothelial corneal dystrophy causing females to be more affected. *Proc Natl Acad Sci USA*. 2020; 117: 573–583. 31852820 10.1073/pnas.1912546116 PMC6955350

