# Supplementary material for: Generation of a Mouse Model of Fuchs Endothelial Corneal Dystrophy by Knock-in of CTG Trinucleotide Repeat Expansion in the TCF4 Gene
Source: Invest Ophthalmol Vis Sci. 2025 Jun 4;66(6):18. doi: 10.1167/iovs.66.6.18 (PMC12151262; doi:10.1167/iovs.66.6.18)
Supplement: Supplement 2 [file iovs-66-6-18_s002.pdf]

Supplementary Table1. The list of primer sequences used to generate KI mice and genotyping

| Method | Target | Strand | Sequence (5'→3') | Name in Fig | Annealing condition | Elongation condition | Band size (bp) |
|---|---|---|---|---|---|---|---|
| Targeting vector | *Tcf4* KI 5' arm | Forward | TTGGGCCCGGGCACAGCGCACTTTGCCAATGGG | | | | |
| | | Reverse | TTGATATCTGGGGGAGCCCCACTTCGAA | | | | |
| | *Tcf4* KI 3' arm | Forward | TTGGATCCCGCTGCTCCTGCTCCACTGCCTGCC | | | | |
| | | Reverse | TTGCGGCCGCCCATAGCTGACAGACAAACACCCTAGC | | | | |
| gRNA | *Tcf4* | Forward | CAGTGGAGCAGGAGCAGCGT | gRNA | | | |
| Genotyping | *Tcf4*$^{KI/KI}$ | Forward | CCATGTCTGCACGCAGAGTG | Fw | 60℃ | 72 ℃ | WT: 1144 |
| | | Reverse | CCTCGGGTGACTTAAACAGT | Rv | 30 sec | 90 sec | KI: 1456 |
