# Supplementary material for: Generation of a Mouse Model of Fuchs Endothelial Corneal Dystrophy by Knock-in of CTG Trinucleotide Repeat Expansion in the TCF4 Gene
Source: Invest Ophthalmol Vis Sci. 2025 Jun 4;66(6):18. doi: 10.1167/iovs.66.6.18 (PMC12151262; doi:10.1167/iovs.66.6.18)
Supplement: Supplement 1 [file iovs-66-6-18_s001.pdf]

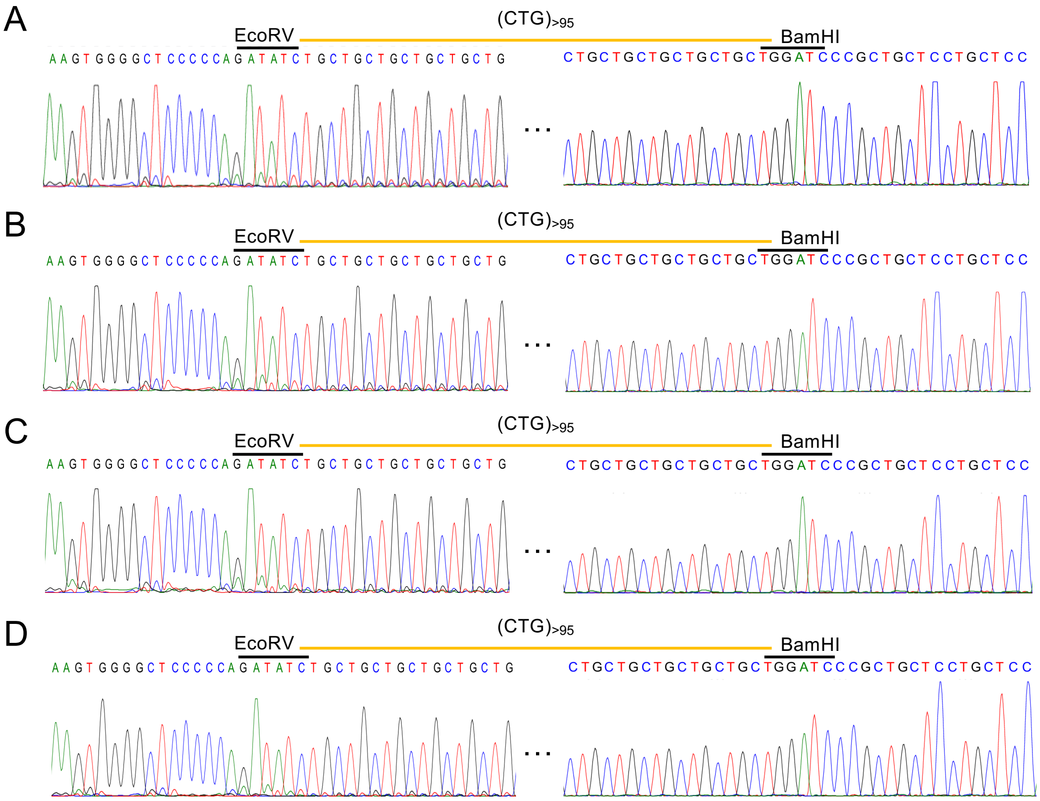

Supplementary Figure 1. Stability assessment of CTG repeat expansion in *Tcf4*$^{(CTG)100/(CTG)100}$ mice at 20 and 60 weeks of age

Sanger sequencing analysis was performed to examine CTG repeat expansion between EcoRV and BamHI restriction sites using genomic DNA extracted from tail biopsies of *Tcf4*$^{(CTG)100/(CTG)100}$ mice. PCR products were purified using the Wizard® SV Gel and PCR Clean-Up System (Promega Corporation, Madison, WI, USA). Sequence chromatograms from independent clones are shown for 20-week-old (two females; A, B) and 60-week-old mice (one male and one female; C: male, D: female). Sequence analysis revealed that all clones maintained more than 95 CTG repeats with precise repeat integrity. Yellow bars indicate CTG repeat regions, and black horizontal bars denote restriction enzyme recognition sites.
